# Synthetic CO_2_-fixation enzyme cascades immobilized on self-assembled nanostructures that enhance CO_2_/O_2_ selectivity of RubisCO

**DOI:** 10.1186/s13068-017-0861-6

**Published:** 2017-07-06

**Authors:** Sriram Satagopan, Yuan Sun, Jon R. Parquette, F. Robert Tabita

**Affiliations:** 1 Department of Microbiology, The Ohio State University, 484 West 12th Avenue, Columbus, OH 43210-1292 USA; 2 Department of Chemistry and Biochemistry, The Ohio State University, 100 West 18th Avenue, Columbus, OH 43210-1185 USA

**Keywords:** CO_2_ fixation, Cell-free systems, Nanostructures, RubisCO, Pathway engineering, Bioconversion

## Abstract

**Background:**

With increasing concerns over global warming and depletion of fossil-fuel reserves, it is attractive to develop innovative strategies to assimilate CO_2_, a greenhouse gas, into usable organic carbon. Cell-free systems can be designed to operate as catalytic platforms with enzymes that offer exceptional selectivity and efficiency, without the need to support ancillary reactions of metabolic pathways operating in intact cells. Such systems are yet to be exploited for applications involving CO_2_ utilization and subsequent conversion to valuable products, including biofuels. The Calvin–Benson–Bassham (CBB) cycle and the enzyme ribulose 1,5-bisphosphate carboxylase/oxygenase (RubisCO) play a pivotal role in global CO_2_ fixation.

**Results:**

We hereby demonstrate the co-assembly of two RubisCO-associated multienzyme cascades with self-assembled synthetic amphiphilic peptide nanostructures. The immobilized enzyme cascades sequentially convert either ribose-5-phosphate (R-5-P) or glucose, a simpler substrate, to ribulose 1,5-bisphosphate (RuBP), the acceptor for incoming CO_2_ in the carboxylation reaction catalyzed by RubisCO. Protection from proteolytic degradation was observed in nanostructures associated with the small dimeric form of RubisCO and ancillary enzymes. Furthermore, nanostructures associated with a larger variant of RubisCO resulted in a significant enhancement of the enzyme’s selectivity towards CO_2_, without adversely affecting the catalytic activity.

**Conclusions:**

The ability to assemble a cascade of enzymes for CO_2_ capture using self-assembling nanostructure scaffolds with functional enhancements show promise for potentially engineering entire pathways (with RubisCO or other CO_2_-fixing enzymes) to redirect carbon from industrial effluents into useful bioproducts.

**Electronic supplementary material:**

The online version of this article (doi:10.1186/s13068-017-0861-6) contains supplementary material, which is available to authorized users.

## Background

The rapid decline of fossil fuel reserves, emission of greenhouse gases, and potential deleterious effects on the biosphere have been highly publicized. Thus there is a considerable interest in designing strategies to capture atmospheric CO_2_ to meet society’s fuel and material needs [1–4]. Clearly, usage of fossil reserves is unlikely to be eliminated any time soon, however, recent advances in industrial biotechnology and increasing usage of biochemical paradigms has provided access to a multitude of starting materials that may be potentially utilized in innovative, economical, and sustainable ways to generate both natural and unnatural value-added compounds [5–7]. Enzymes have evolved over billions of years into efficient and highly selective catalysts of chemical reactions, particularly those that are energetically unfavorable under ambient conditions, facilitating their role in the maintenance of life on this planet [8]. Although advances have been made to synthesize products using engineered in vivo biological systems, sustainability, and scale-up capabilities are often limited by the tug-of-war between the cell’s objectives and the desired product output. Cell-free biomimetic systems have smaller ecological footprints and are convenient alternatives with a potential for rapid design-build-test cycles [5, 8, 9]. Sequential chemical conversions are often achieved in these systems via multienzyme cascades that are spatially organized into three-dimensional structures, which control intermediate flux and enhance overall catalytic efficiency [10–12]. Multienzyme cascades have been constructed on synthetic scaffolds via covalent linkages or within physical compartments to proximally position the enzymes to channel reaction intermediates from one catalytic site to the other [13–19]. However, most of these strategies require a significant synthetic effort to implement and few control three-dimensional structure at the nanoscale. Developments in site-specific protein-nanoparticle conjugation techniques and DNA nanotechnology have enabled the spatial arrangement of proteins into arrays [20–22]. Alternatively, the self-assembly of small molecules offer an expedient strategy to create nano-structured scaffolds to support enzymatic arrays [23–30]. Block copolymers, nanotubes, and DNA nanocages have been used to create nanoscale supports for enzymes [31–34]. However, the potential to co-assemble carbon fixation and associated assimilatory pathway enzymes into nanostructured catalytic platforms remain untested.

RubisCO is the world’s most abundant protein and accounts for most of the biological CO_2_ fixed on earth. Diverse structural forms of RubisCO were previously characterized, with varying catalytic properties, stabilities, and temperature/pH optima noted [35]. Complex assembly requirements and sensitivity to a variety of inhibitors, effectors, and denaturing conditions have been impediments to the design of stable catalytic platforms using RubisCO. In fact, reconstituting RubisCO activity from inactive and not fully assembled forms requires the action of chaperone and other specialized proteins [36–41]. It has also been a challenge to assemble functional RubisCO into stable, scalable host cells to capture CO_2_ for various applications, including improvement of the host cell’s primary productivity [42]. Cyanobacteria and some proteobacteria optimize CO_2_ fixation by encapsulating RubisCO and carbonic anhydrase (CA) within intracellular compartments called carboxysomes; analogous micro-compartments are employed by eukaryotic algae [43, 44]. In a recent study, functional RubisCO was co-encapsulated with CA in synthetic carboxysome mimics, which could protect the enzyme from proteolytic degradation [45]. With these biological structures in mind, we postulated that RubisCO and other enzymes might be coaxed to self-assemble with nanostructures. Starting with simple di- or tetra-peptide compounds capable of self-assembling into nanotubes or nanofibers, we show here that structurally divergent RubisCO enzymes can form CO_2_-fixing nanostructure-enzyme complexes. As a proof of concept for utilizing these nanostructures to assemble entire pathways, we further demonstrated that RubisCO could be coupled with enzymes that catalyze sequential steps of the CBB cycle or the pentose-phosphate pathway within the same nanostructures. Furthermore, the immobilization of one form of RubisCO used in these studies resulted in a significant increase (compared to the unbound enzyme) in the CO_2_/O_2_ specificity factor, the largest enhancement in specificity of RubisCO thus far achieved.

## Results

### Nanostructure assembly and optimization of recovered activities using RubisCO

**Fig. 1:**
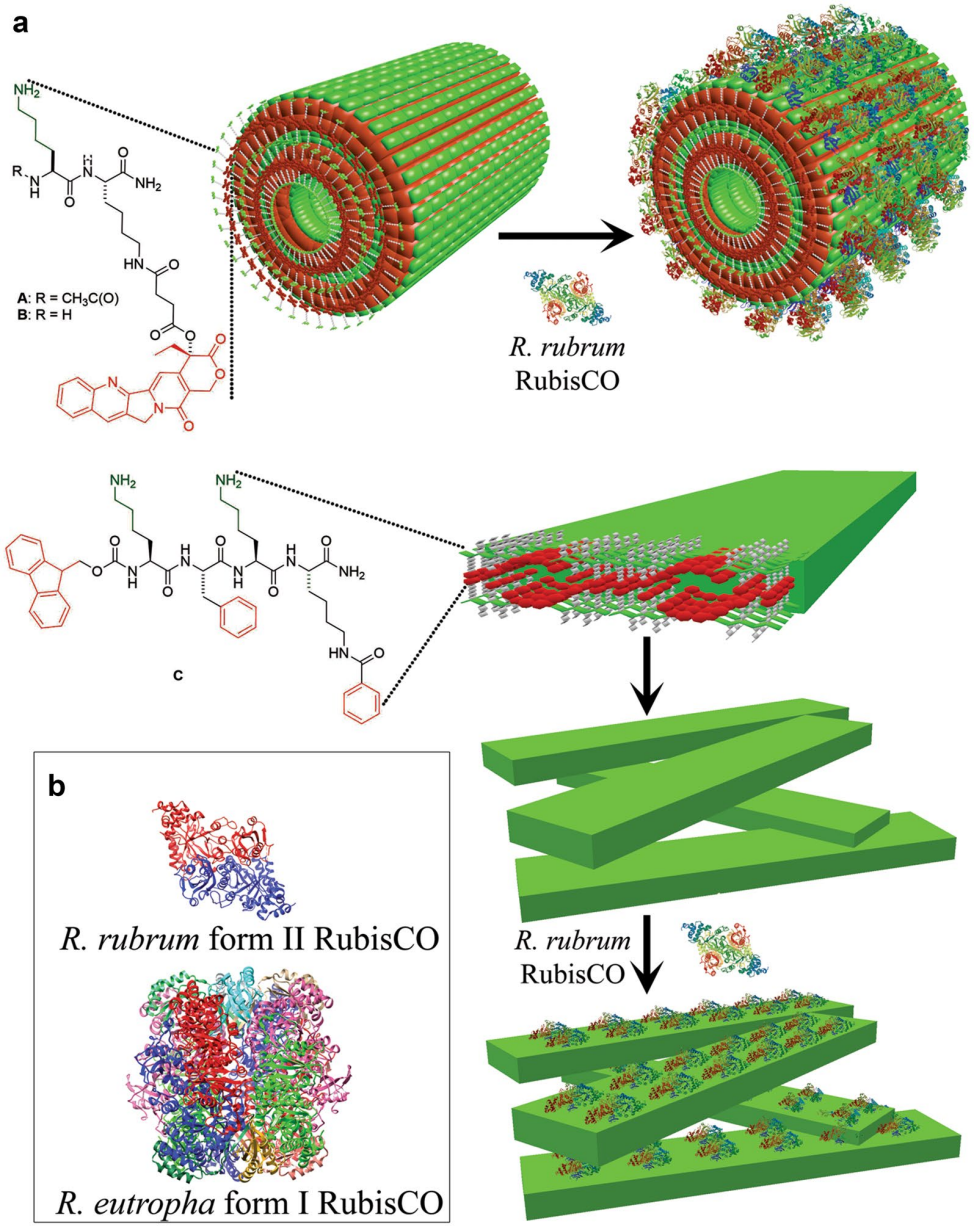
Illustration of the assembly of nanostructures with RubisCO driven by electrostatic interactions. **a** Structures of compounds that self-assemble into either nanotubes (**A**/**B**) or nanofibers (**C**). **b** Either the dimeric *R. rubrum* form II RubisCO (PDB id—5RUB) or the hexadecameric *R. eutropha* form I RubisCO (PDB id—1BXN) were used in these studies

Initial studies were performed by mixing RubisCO (0.1 mg) with a previously described [46] bola amphiphilic naphthalene diimide (NDI-Lys) self-assembling monomer (1 mM) in 1 mL of a buffered solution. These experiments resulted in the precipitation of RubisCO. Part of the incompatibility of RubisCO with these nanotubes arose from the buffered systems necessary to stabilize RubisCO, which screened the charged nanotube head groups, leading to uncontrolled aggregation and precipitation. Thus, two small dipeptide conjugates (compounds **A**/**B**) and a fluorenylmethyloxycarbonyl (Fmoc) tetrapeptide (compound **C**) were chosen based on their ability to self-assemble into stable nanotubes and nanofibers, respectively, with RubisCO in a Bicine buffer (Fig. 1a). Consistently more than 20% of the RubisCO activity present in the unbound enzyme sample could be recovered from these nanostructure-RubisCO complexes. The greater surface charge of the dipeptide monomers, owing to the lysine side-chains, attenuated assembly/aggregation in buffered solvents leading to well-dispersed self-assembled structures. We have previously demonstrated that the dipeptide derivatives (**A**/**B**) self-assemble into nanotubes with diameters ranging from 80 to 120 nm under buffered conditions (Fig. 1a) [26]. Similarly, short peptides, such as **C**, form amyloid-type β-sheet structures that afford stable, one-dimensional nanofibers with 20–30 nm diameters at concentrations as low as 1 mM (Fig. 1a) [47]. Variants of compound **A** and **B** with only one Lys or with the second Lys replaced by either Arg or Glu were also predicted to form similar nanotubes (Fig. 1a). However, when mixed with RubisCO, most of these variants resulted in precipitation and significant loss of RubisCO activity. The mono-lysine variant of compound B could allow for nanostructure formation with RubisCO, but only 7% of the starting level of RubisCO activity was recovered from this complex. Another compound with predominantly acidic amino-acids conjugated to a 7-(diethylamino)-3-coumarin carboxylic acid (Fmoc-EFEK-DAC) also resulted in complete precipitation and loss of RubisCO activity upon mixing. Thus, among various compounds tested for nanostructure-complex formation with RubisCO, compounds **A**, **B**, and **C** seemed most compatible and resulted in complexes with significant RubisCO activity recoveries.

Two structural forms of RubisCO were employed in these studies: the simple form II L_2_ dimer RubisCO from *Rhodospirillum rubrum*, which has an elliptical shape with approximate dimensions of 5 × 7 × 10 nm, and the L_8_S_8_ hexadecameric form I enzyme from *Ralstonia eutropha* that is shaped like a cube with approximately 10 nm sides (Fig. 1b) [48–50]. Accordingly, the dimensions of the nanotubes and nanofibers should readily accommodate both forms of RubisCO. Initial experiments indicated that co-assembly of dipeptides **A** or **B** and the enzyme resulted in low yields of activity in the nanotube-form II RubisCO complexes (equivalent to 0.01–0.05 mg RubisCO per mg nanotube). Further, different preparations of nanotubes co-assembled from different batches of compounds **A** or **B**, and RubisCO resulted in widely different activity recoveries (equivalent to 0.005–0.12 mg of RubisCO per mg nanotube), despite starting with identical concentrations of the constituents.

Although compounds **A** and **B** had been prepared following identical procedures that resulted in highly pure compounds, and stored as lyophilized samples, TEM images of monomer samples from different preparations indicated that some of the older preparations had varying amounts of pre-assembled nanotubes (formed during storage as lyophilized powders) relative to newer preparations (Additional file 1: Figure S1a). Higher RubisCO activity recoveries were typically obtained with these older samples, suggesting that the nanotube precursors (i.e., monomeric compounds **A** or **B**) inhibited the enzyme activity during the co-assembly process. RubisCO activity assays performed by mixing the unbound enzyme with either of the monomeric compounds (fresh preparations), or with fully assembled nanotubes that had been isolated using ultracentrifugation confirmed the inhibitory effect of monomer compounds **A** and **B** (Additional file 1: Figure S1b). Because the pre-assembled nanotubes did not seem to significantly affect the activity of unbound RubisCO, it proved advantageous to assemble enzymes with pre-formed and isolated nanostructures rather than co-assembling them in the presence of monomeric compounds. Indeed, consistently higher recoveries of enzymatic activities (equivalent to 0.1–1 mg RubisCO per mg nanostructure) were obtained by mixing the enzyme with pre-formed nanostructures that had been isolated from unassembled monomers via ultracentrifugation. Re-suspension or washing the nanostructures with the Bicine buffer in the presence or absence of up to 300 mM sodium chloride did not result in any loss of activity, indicating that the enzyme was not weakly bound to the structures (Additional file 1: Table S1).

**Fig. 2:**
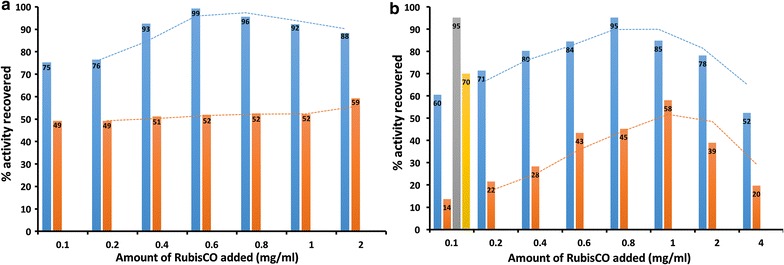
RubisCO loading with fixed amounts of pre-assembled nanotube **A** (**a** 0.75 mg/mL) or nanofiber **C** (**b** 0.9 mg/mL). Plots show activities measured from nanostructure-RubisCO complexes that had been loaded with varying amounts of either form I (*blue*) or form II (*orange*) RubisCO enzymes. Recovery percentages were calculated relative to the enzyme activities in the corresponding samples with unbound RubisCO. Activities were also measured from identical form I (*gray*) or form II (*yellow*) nanofiber **C** preparations (**b**) that had been supplemented with 1 mg/mL bovine serum albumin (BSA) during RubisCO loading

A range of 1–20 mM of the monomeric compounds **A**–**C** were used in the initial experiments for formation of the respective nanostructures, which were then isolated from unassembled monomers using ultracentrifugation. For each preparation, an appropriate volume of the nanostructure resuspension was mixed with either form I or form II RubisCO in Bicine buffer, resulting in a 2.5- to 5-fold dilution of the nanostructure suspensions in the final sample. Optimal recovery of enzymatic activity for nanotube-RubisCO complexes was obtained with nanotubes prepared from 10 to 20 mM of monomeric compounds (**A** or **B)**, for form I and form II enzymes, respectively. The optimal starting concentration of compound **C** for the formation of nanofiber-RubisCO complexes was 2.5 and 5 mM for form I and form II RubisCO, respectively (Additional file 1: Table S2). Lower starting concentrations of nanostructure materials typically resulted in lower yields of the nanostructure-RubisCO complexes, as discernible from the sizes of pellets upon ultracentrifugation. This explains the lower rates of activity recoveries in these cases (Additional file 1: Table S2). Loading experiments were performed by titrating different amounts of RubisCO with fixed amounts of nanomaterials to determine optimal ratios for immobilization. RubisCO concentrations in these initial experiments were in the range 0.1–4 mg/mL in the final preparations. However, for both form I and form II RubisCO enzymes, using more than 2 mg/mL in the final preparation resulted in reduced enzyme activity recovery (Fig. 2). This could be attributed to enzyme precipitation, which resulted in the formation of pellets even with a low-speed centrifugation (8000*g* for 5 min at 4 °C). Notably, no RubisCO remained unassembled at loading concentrations less than or equal to 1 mg/mL, as indicated by the absence of protein or activity in the supernatants recovered after ultracentrifugation. Thus, the lower activity recovery at these concentrations likely reflects the limited surface availability of RubisCO active sites for substrate diffusion in these nanostructures. The addition of a non-specific protein, i.e., bovine serum albumin (BSA), to the samples with lower RubisCO loads resulted in higher activity recoveries typically observed with higher RubisCO loads (Fig. 2). In addition to its well-perceived role in stabilizing proteins, especially in dilute solutions, the smaller size (66.5 kDa, monomer) and the lower pI (4.7) of BSA likely results in displacement of RubisCO molecules from less accessible locations on the nanostructures, making more active sites accessible for activity determination.

### Visual characterization of nanostructure-enzyme complexes

**Fig. 3:**
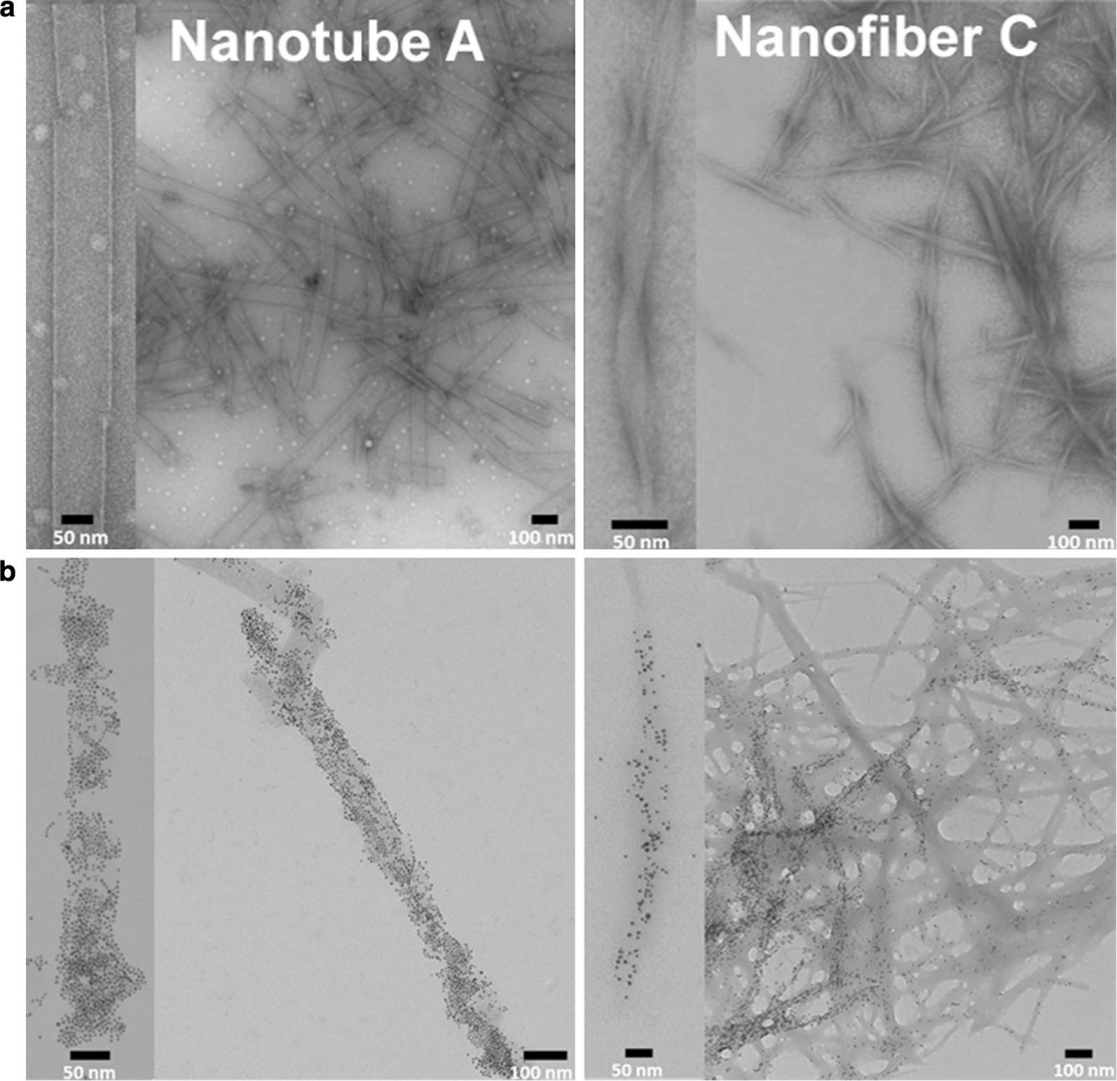
TEM images of nanostructures. **a** Nanotubes and nanofibers formed from compounds **A** (*left*) and **C** (*right*), respectively, stained with uranyl acetate. **b** TEM images of Ni–NTA Nanogold^®^ particles bound to the hexa-histidine tagged form I RubisCO and associated with either nanotube **A** (*left*) or nanofiber **C** (*right*). Images are representative of multiple samples imaged from independent preparations. For better clarity, a close-up view of a single nanostructure is shown to the *left* of each image

Transmission electron microscopy (TEM) was used to visually characterize the nanostructures. Dipeptides **A** and **B** formed uniform cylindrical open-ended nanotubes and compound **C** assembled into a network of β-sheet nanofibers (Fig. 3a). The distribution of enzyme molecules associated with the nanostructures was assessed by TEM using nanogold particles that were specifically bound to the enzymes. This was facilitated by the presence of multiple nickel-nitrilotriacetic acid functionalities on the nanogold particles that formed stable, high-resolution complexes with amino-terminal hexa-histidine tags of the recombinant microbial enzymes used in this study. After assembly, these nanostructure-enzyme complexes were visualized by standard TEM without the need for staining or signal enhancement. The density of visible nanogold spots clearly showed that the enzymes were bound to the surface of the nanotubes formed by **A**/**B** and the nanofibers formed by **C** (Fig. 3b).

### In-situ production of RuBP within nanostructures using a cascade of enzymes

**Fig. 4:**
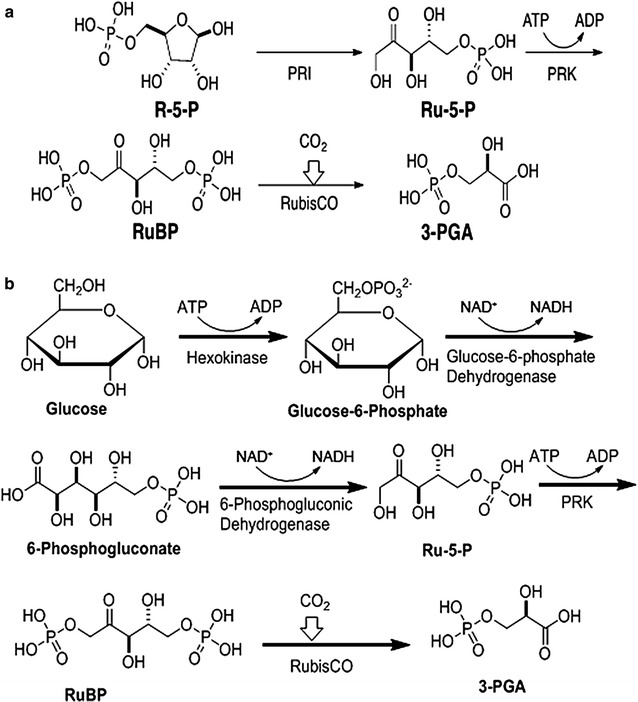
Schematic of CO_2_-fixation pathways assembled in nanostructures. Cascade of enzymatic steps employed to convert either ribose-5-phosphate (R-5-P) (pathway 1 **a**) or glucose (pathway 2 **b**) to 3-PGA

To establish a nanostructure-supported multienzyme array, we co-assembled enzymes that catalyze additional reactions in either the CBB cycle for CO_2_ fixation (pathway 1, Fig. 4a) or the pentose phosphate pathway (pathway 2, Fig. 4b). The partial CBB-pathway reconstruction was accomplished by co-binding the enzymes phosphoriboseisomerase (PRI) and phosphoribulokinase (PRK) to the nanostructures, which would sequentially catalyze the conversion of ribose-5-phosphate (R-5-P) to ribulose-5-phosphate (Ru-5-P), and then to RuBP, with RuBP serving as the substrate for RubisCO in the nanostructures, resulting in the formation of 2 molecules of 3-phosphoglyceric acid (3-PGA) (Fig. 4a). The enzymes PRK and PRI were each added at a concentration of 0.1 mg/mL along with 1.0 mg/mL RubisCO for assembly into nanostructures. Using higher concentrations of PRK or PRI did not improve the reaction flux because the activity of RubisCO was rate-limiting. Pathway 2 (Fig. 4b) was designed to utilize glucose, a simpler and an industrially relevant feedstock, as the starting substrate. This involved the conversion of glucose to Ru-5-P utilizing the enzymes hexokinase (added at 0.05 mg/mL), glucose-6-phosphate dehydrogenase (added at 0.1 mg/mL), and 6-phosphogluconate dehydrogenase (added at 0.7 mg/mL) from the pentose phosphate pathway, and the subsequent conversion of Ru-5-P to 3-PGA using PRK (added at 0.05 mg/mL) and form II RubisCO (added at 0.4 mg/mL) (Fig. 4b). Because bacterial forms of all three CBB enzymes were used in engineered pathway 1, it was possible to express the genes (in *Escherichia coli*) and purify the resultant recombinant proteins containing *N*-terminal hexa-histidine tags, allowing for specific binding with nanogold particles for TEM imaging. The nanostructures associated with either nanogold-PRK or with both RubisCO and PRK each conjugated to different nanoparticles, all resulted in a nanogold-spot distribution pattern that was comparable to what had been observed with just RubisCO in the nanostructures (Additional file 1: Figure S2; Fig. 3). It is thus likely that the overall morphology of the nanostructure-enzyme complexes did not change in response to the type or number of enzymes associated.

Specific formation of stoichiometric amounts of 3-PGA in these reactions was confirmed and quantified using commercial enzymes that couple 3-PGA reduction to glyceraldehyde-3-phosphate formation with the concomitant oxidation of NADH, which could be followed spectrophotometrically. Also, the activities of individual enzymes bound to the nanostructures could be determined in independent assays utilizing the same nanostructure-enzyme complexes (Additional file 1: Table S3). The overall flux through all three enzymatic steps in pathway 1 ranged from 63 to 97% for the nanostructure-enzyme complexes relative to what was measured using an identical mixture of unbound enzymes (Table 1). Similarly, the pathway fluxes were also determined for the conversion of glucose to 3-PGA (Table 2). Because the steps in pathway 2 resulted in acidification of the reaction mixture, a higher buffer concentration was used. The activity of commercial 6-phosphogluconic dehydrogenase was found to be rate-limiting for this pathway. Although the overall glucose to 3-PGA pathway flux was much lower for the enzyme cascade in both the unbound and the nanostructure-bound samples (Table 2), it can be optimized further by controlling the pH of the reaction mixture and by using enzymes with higher specific activities. Furthermore, two-enzyme nanostructures containing both PRK and RubisCO in the same complex or as separate single-enzyme nanostructure preparations with only RubisCO or PRK, when subsequently mixed, both resulted in combined sequential activity (i.e., the conversion of Ru-5-P to RuBP and then to 3-PGA) (Additional file 1: Table S4). These observations suggest that different sets of enzymes associated with dissimilar nanostructures could be combined as separate interconnected modules to generate specific products.

**Table 1:** Activities measured for the conversion of R-5-P to 3-PGA in nanostructure-multienzyme complexes with *R. eutropha* form I (FI) or *R. rubrum* form II (FII) RubisCO, PRK and PRI

Enzyme cascade	Combined activity^a^ (μmoles/min-mg)	% Activity ret.^b^
Unbound FI/PRK/PRI	4.08	100
FI/PRK/PRI-nanotube **A**	3.39	83
FI/PRK/PRI-nanotube **B**	3.49	86
FI/PRK/PRI-nanofiber **C**	3.94	97
Unbound FII, PRK and PRI	3.03	100
FII/PRK/PRI-nanotube **A**	2.45	81
FII/PRK/PRI-nanotube **B**	2.52	83
FII/PRK/PRI-nanofiber **C**	1.90	63

^a^Calculated from stable ^14^CO_2_ fixed

^b^Activities retained are presented relative to an unbound enzyme mixture comprising all three enzymes. Activity numbers are from one of two independently prepared and assayed nanostructure complexes that yielded similar results

**Table 2:** Activities measured for the conversion of glucose to 3-PGA in nanostructure-multienzyme complexes with *R. rubrum* form II (FII) RubisCO

Enzyme cascade	Combined activity^a^ (μmoles/min-mg)	% Activity ret.^b^
Unbound FII and glucose pathway^c^	0.259	100
FII and glucose pathway^c^ in nanotube **A**	0.100	39
FII and glucose pathway^c^ in nanotube **B**	0.110	42
FII and glucose pathway^c^ in nanofiber **C**	0.115	44

^a^Calculated from stable ^14^CO_2_ fixed

^b^Activities retained are presented relative to an unbound enzyme mixture comprising all enzymes. Activity numbers are from one of the two assays conducted with independently prepared and assayed nanostructure complexes, which gave similar results

^c^Glucose pathway comprises all enzymes that constitute pathway 2

### Protease protection of nanostructure-associated enzymes

**Fig. 5:**
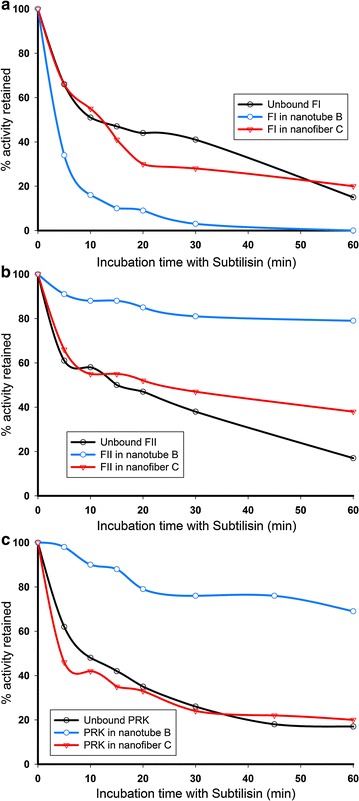
Proteolytic sensitivities of RubisCO and PRK enzymes in nanostructure complexes. Unbound or nanostructure-associated RubisCO (**a**, **b**) or PRK (**c**) were treated with subtilisin for various times and residual enzymatic activities were measured for each time point. Data shown here is representative of two independent preparations that gave similar results. Nanotubes **A** or **B** provided identical levels of protection to all enzymes and hence the data is shown for only one of them (i.e., nanotube **B**), along with the data for nanofiber **C**

The accessibility of the nanostructure-bound enzymes was evaluated by treating the co-assembled structures with a protease (subtilisin). Whereas, none of the nanostructures could protect the larger, more complex form I enzyme from inactivation and presumably proteolytic degradation (Fig. 5a), high levels of activity were retained by the subtilisin-treated nanotube complexes of the structurally simpler form II RubisCO or PRK enzymes (Fig. 5b, c). The larger form I RubisCO likely assembles with the nanostructures via weaker surface interactions or with a conformation that results in about the same (in the case of nanofiber **C**) or an even greater accessible surface area of the enzyme that is exposed to protease. However, the smaller sizes of the form II RubisCO and PRK enzymes likely ensures that the bulk of the enzyme surface is sequestered via interactions with the nanotube, thereby protecting these smaller proteins from proteolysis. Owing to the dimensions, nanofibers tend to have a greater area exposed relative to the nanotubes, which might increase the susceptibility of associated proteins to proteolytic degradation.

### Enzymatic properties of nanostructure-bound RubisCO

**Fig. 6:**
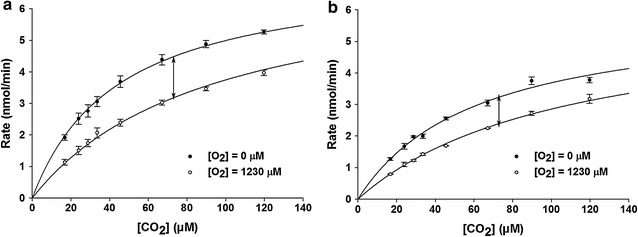
Reaction velocity measurements of *R. eutropha* form I RubisCO as a function of CO_2_ concentration in the presence (*open circles*) or absence (*closed circles*) of saturating levels of oxygen (i.e., 1230 μM). **a** Michaelis–Menten curves for carboxylation activities measured with unbound *R. eutropha* form I RubisCO. **b** Michaelis–Menten curves for carboxylation activities measured with nanotube **B**-form I RubisCO complexes. The extent of oxygen inhibition for each enzyme preparation is indicated (*double*-*headed arrows*). The *K*_O_ (*K*_I_) for O_2_ was enhanced about 1.6-fold for the nanotube **B**-form I enzyme complex compared to the unbound enzyme (see Table 3 for kinetic constants). The average value and *error bar* for each data point was calculated with values obtained from two independent assays (with different preparations) performed with identical CO_2_ concentrations

The lower levels of activity exhibited by RubisCO typically limits and determines the CBB pathway flux [35]. Several studies have shown that important properties of various enzymes might be enhanced via immobilization or sequestration of some enzymes [33, 34]. Thus, it was of interest to determine if the association with nanostructures could alter the catalytic properties of RubisCO, the rate-limiting enzyme for global biological CO_2_ fixation. Indeed, the association with nanostructures caused increases in the substrate *K*_m_ values (*K*_c_ for CO_2_ and *K*_o_ for O_2_) of the form I enzyme (Table 3). The higher increase in *K*_o_ values relative to *K*_c_ values resulted in significantly higher CO_2_/O_2_ specificity factors (*Ω*) for the form I enzyme that was associated with the nanostructures (Table 3; Fig. 6 and Additional file 1: Figure S3). Immobilization did not seem to significantly perturb the *K*_c_, *K*_o_, or the *Ω* values of the nanostructure-associated form II RubisCO (Table 3). Because the enzymes’ *k*_cat_ values were determined based on the amount of enzyme added to the nanostructures, the values obtained for the nanostructure-bound enzymes were generally lower, reflecting the lower rates of enzyme accessibilities and activities recovered (Additional file 1: Table S3c; Table 3).

**Table 3:** Catalytic properties of RubisCO enzymes (*R. eutropha* form I (FI) and *R. rubrum* form II (FII) associated with nanostructures

Enzyme^a^	*Ω* ^b^ (*V*_c_ *K*_o_/*V*_o_ *K*_c_)	*k*_cat_^b^ (sec^−1^)	*K*_c_^b^ (μM)	*K*_o_^b^ (μM)	*K*_o_/*K*_c_^c^
Unbound FI	79 ± 1	2.6 ± 0.5	41 ± 4	1093 ± 141	27
FI-nanotube **B**	95 ± 3^d^	1.8 ± 0.1^e^	53 ± 7	1756 ± 341	33
FI-nanofiber **C**	88 ± 1^d^	2.2 ± 0.1^e^	56 ± 8	1693 ± 383	30
Unbound FII	17 ± 2	1.2 ± 0.1	100 ± 7	96 ± 26	1.0
FII-nanotube **B**	17 ± 1	0.6 ± 0.2^e^	120 ± 24	170 ± 41	1.4
FII in nanofiber **C**	17 ± 1	0.6 ± 0.1^e^	96 ± 16	95 ± 32	1.0

^a^Association with either of the nanotubes (i.e., **A** or **B**) imparted similar properties to both form I (FI) and form II (FII) enzymes

^b^Values are the mean ± standard deviation (*n*−1) of at least three independent nanostructure-enzyme complex preparations

^c^Calculated values

^d^An unpaired *t* test with *Ω* values obtained from three independent experiments gave p values of 0.0006 and 0.0003 for FI-nanotube **B** and FI-nanofiber **C**, respectively, relative to unbound FI

^e^These *k*_cat_ values were calculated based on the initial amounts of enzyme added to constitute nanostructure-RubisCO complexes. They are not significantly different from the value measured with the unbound enzyme, if the percentage recoveries are factored into the calculations (Additional file 1: Table S3c) for each experiment

## Discussion

Rising greenhouse gas emissions have prompted chemists, biologists, and engineers alike to explore strategies for the capture of atmospheric CO_2_ and its conversion to useful products and biofuels [1, 4, 51–53]. Enzymes offer high fidelities for chemical conversions albeit being limited by structural stabilities and other metabolic constraints when operating in biological cells [5, 8]. Thus, exploring ways to assemble enzymes as part of cell-free biocatalytic systems has become a widespread endeavor, with an aim to engineer directed pathways for producing products of interest. Synthetic short peptide-based amphiphiles are promising precursors that self-assemble into diverse nanostructured materials. Simple and straight-forward procedures for synthesis, biodegradability, and modularity makes them uniquely suited for a multitude of applications. Self-assembling peptides have been used as scaffolds for tissue engineering, drug delivery, gene delivery, biosensing, and biomolecular signaling [30]. Our study demonstrated the utility of these scaffolds for CO_2_-capture applications, which is likely to aid industrial strategies for carbon capture.

Enzyme immobilization in these scaffolds seems to occur via weak charge-based associations with the nanostructures. All the scaffolds that could functionally assemble with the enzymes carry a net positive charge at pH 8 used in these experiments. Correspondingly, the pI values of all the enzymes used in this study are less than 8. Using similar scaffolds with no net charge or those with net negative charge precluded functional assembly with enzymes. This generic charge-based self-assembly also allowed easy addition of multiple enzymes at desired loading ratios to the same scaffold. TEM images of gold-nanoparticle-tagged enzymes immobilized into these scaffolds provided visual proof of the enzymes present in these complexes (Fig. 3). The ability to assemble at least two different enzyme cascades into the same set of scaffolds demonstrated the applicability of this system for assembling larger pathways. Because enzymes present in multiple scaffolds could be brought together in assays for obtaining combined sequential activities (Additional file 1: Table S4), it should be possible to spatially separate charge-incompatible enzymes that are part of a pathway by placing them on different scaffolds and bringing them together during assays.

None of the nanostructures could impart improvements to thermal stability for any of the enzymes (data not shown), and smaller enzymes could be protected from proteolysis when associated with nanotubes (Fig. 5). The weak charge-based association that drives both the nanostructure assembly and the formation of nanostructure-enzyme complexes explains the temperature-instability of these complexes. Employing novel composites to capture nanostructures into higher order polymers [52] or the use of crosslinking [54] are potential strategies that could lead to further stabilization of the nanostructure-enzyme complexes. Protection from proteolysis could be attributable to a higher surface packing density and better sequestration of smaller enzymes associated with nanotubes. Presumably, a proportion of the sequestered form II RubisCO or PRK enzyme molecules retain accessibility to substrates and products (i.e., for activity measurements) despite being resistant to proteolytic cleavage. It is intriguing that the nanotube-associated form I enzyme is more susceptible to proteolytic cleavage when compared with the unbound enzyme (Fig. 5a). Whereas the access for subtilisin to unbound form I RubisCO molecules is diffusion controlled, the association with nanotubes and a lower surface packing density likely presents easy surface-access for subtilisin. The differences in surface packing densities may also explain the dissimilar pattern of form I versus form II RubisCO loading onto nanotubes (Fig. 2a). The protective effect is likely diminished for all enzymes associated with nanofiber **C** because the peptide sequence in compound **C** likely results in partial proteolytic degradation of the nanofiber itself (Fig. 5). Although protection from proteolysis seems irrelevant to industrial scale up applications, our results provide a good measure of enzyme accessibility to other external agents that may be pertinent to specific applications.

A substantial and desirable outcome of immobilizing RubisCO in the nanostructures is the increased *K*_o_ of the form I enzyme, which leads to an increased CO_2_/O_2_ specificity factor (*Ω*) (Fig. 6; Table 3 and Additional file 1: Figure S6). This is the most significant enhancement of specificity factor thus far reported for any source of RubisCO and greatly exceeds specificity enhancements achieved via mutagenesis and bioselection [55–63]. Moreover, there was little or no compensatory decrease in the *k*_cat_ for the nanostructure-associated enzyme as often occurs with specificity-enhanced soluble mutant RubisCOs [58, 59]. The apparent increases in the *Ω* value of nanostructure-associated form I RubisCO is presumably a result of the altered electrochemical microenvironment surrounding the larger RubisCO molecules, which could result in preferentially occluding the paramagnetic O_2_ (relative to CO_2_) from entering RubisCO’s active site. This would also explain the observed increases in the *K*_o_/*K*_c_ ratios of the nanostructure-associated form I RubisCO (Table 3). Because the increases in *Ω* value is only observed with the form I RubisCO and not form II RubisCO, it must be concluded that this is not a result of preferential O_2_-occlusion by these nanostructures. Small subunits are known to concentrate RubisCO’s catalytic large subunits via ionic and hydrogen-bond interactions involving polar and charged surface residues of both subunits. Based on the structural analysis of several form I RubisCO structures, it is evident that amino acid residues involved in these interactions are directly connected to the active site via secondary structural elements. Thus, the conformational differences occurring during catalysis in the form I enzymes are induced and influenced by small subunits [64]. Other subtle structural differences between the form I and form II RubisCOs, including the presence of a central solvent channel in form I enzymes, have been correlated with differences in their substrate binding and a more “open” structure exhibited by the *R. rubrum* form II RubisCO [64]. Concentration of catalytic large subunits via association with small subunits or through other interactions leading to higher order structures like carboxysomes has been correlated to some form of structural and/or functional advantage [65]. In the absence of small subunits in the *R. rubrum* form II enzyme, surface residues that are usually solvent-exposed could be involved in interactions with the nanostructure, leading to a diluted but well-packed nanostructure-RubisCO matrix. The absence of modulatory structural elements and a more “open” conformation likely explain the unaltered *Ω* values measured for the form II enzyme present in these nanostructure complexes. Based on these results, it may be fruitful to systematically analyze the effect of immobilization (onto different scaffolds) on structurally divergent RubisCO enzymes. In addition, modulating the chemical structure of nanostructure-forming monomers to preferentially exclude O_2_ from these complexes may result in further enhancement of RubisCO’s *Ω* value. Likewise, co-immobilization with carbonic anhydrase might further enhance CO_2_ availability around RubisCO molecules as in carboxysomes and analogous micro-compartments [44, 45].

## Conclusions

A new application is described for simple self-assembling peptide-based nanostructures. We have demonstrated that the sequential enzymes that are part of a pathway for CO_2_ utilization could be co-assembled into catalytically active nanotube- and nanofiber-supported multienzyme complexes. Further, the association with nanostructures appears to improve structure–function properties of the enzymes, with a significant enhancement of form I RubisCO’s CO_2_/O_2_ selectivity attained. Given the fact that RubisCO is a key enzyme that accounts for most CO_2_ fixed on earth, these results suggest that stable scaffolds may be prepared to encapsulate structurally complex enzymes along with RubisCO to constitute entire pathways for converting CO_2_ into useful products. Further, the use of simple starting materials such as glucose allows for sampling a wide array of compounds, some of which could eventually be employed with alternate nanostructures possessing properties more conducive to efficient product formation. The use of enzymes from thermophilic organisms and cross-linkable nanostructures might also facilitate scale-up for industrial applications.

## Methods

### Synthesis of peptide conjugates

Peptide conjugates were manually prepared using Fmoc/*t*-Bu solid-phase peptide synthesis on Rink amide resin (loading 0.8 mmol/g). Amide-coupling steps were accomplished with standard techniques for all amino acids: Fmoc-amino acid, 1,3-diisopropylcarbodiimide (DIC), and 1-hydroxybenzotriazole (HOBt) (500 mol% each relative to resin) in 1:1 DMF/DCM for 1.5 h. A solution of 20% piperidine in DMF was used for Fmoc removal and 1% TFA in dichloromethane was used for 4-methyltrityl (Mtt) group de-protection. Peptide conjugates were cleaved from the resin by treatment with TFA/water/triethylsilane (95/1/4) at room temperature for 2 h. Crude peptides were precipitated with cold diethyl ether and purified by reversed-phase HPLC, using a preparative Varian Dynamax C-18 column and eluting with a linear gradient of acetonitrile/water containing 0.1% TFA (10/90 to 100/0 over 30 min). These precipitates were stored as lyophilized powers at 0 °C. Purity was assessed by analytical reverse-phase HPLC and identity confirmed using ESI–TOF mass spectrometry and NMR. All reactions were performed in an atmosphere of argon or nitrogen. ^1^H NMR was recorded at 400 MHz and ^13^C NMR spectra at 100 MHz on a Bruker DPX-400 instrument. Dipeptide conjugates **A** and **B** were synthesized as previously reported [26]. The tetrapeptide conjugate **C** was prepared using a similar procedure in which the ε-amino group of the carboxy-terminal lysine was reacted with benzoic anhydride (500 mol%), DIPEA (500 mol%) in DMF for 24 h (Additional file 1: Figure S4). Purity of the resultant compound was verified by HPLC fractionation (Additional file 1: Figure S5).

### Characterization of Fmoc-KFKK(Bz)-NH_2_ (compound **C**)

^1^H NMR (400 MHz, DMSO-d_6_) δ 8.46–8.43 (m, 1H), 8.21–8.17 (m, 1H), 7.91–7.81 (m, 5H), 7.74–7.64 (m, 6H), 7.54–7.31 (m, 8H), 7.25–7.12 (m, 4H), 7.05–7.02 (m, 1H), 4.60–4.56 (m, 1H), 4.35–4.16 (m, 4H), 3.93–3.88 (m, 1H), 3.29–3.21 (m, 2H), 3.09–3.04 (m, 1H), 2.83–2.67 (m, 5H), 1.72–1.64 (m, 2H), 1.60–1.38 (m, 9H), 1.38–1.24 (m, 4H); ^13^C NMR (100 MHz, DMSO-d_6_) 173.50, 171.63, 171.04, 170.92, 166.11, 158.27, 157.96, 143.84, 143.68, 140.71, 137.49, 134.62, 130.98, 129.24, 128.19, 127.89, 127.63, 127.09, 125.20, 120.12, 120.09, 65.59, 52.32, 46.66, 38.66, 31.84, 31.29, 28.89, 26.57, 22.81, 22.33, 22.32, 22.12; ESI–MS for C_49_H_63_N_8_O_7_ [M+H]^+^ calculated 875.4820; found 875.4825.

### Nanostructure preparation, isolation, and assembly with enzymes

For the preparation of nanostructures, monomeric compounds (**A**, **B**, or **C**) were added to 50 mM Bicine-NaOH, 10 mM MgCl_2_, pH 8.0 (Bicine buffer) at concentrations of 5–20 mM, sonicated for 15 s using a Model W-385 Sonicator (Heat Systems-Ultrasonics, Inc.) and incubated at room temperature for 64–72 h to promote self-assembly. Nanostructure pellets were obtained by ultracentrifugation at 418,000*g* for 1 h at 4 °C and re-suspended to original volumes in Bicine buffer. These pellets were mixed with enzymes, incubated for 16–20 h at 4 °C, re-isolated using ultracentrifugation, and re-suspended in original volumes of Bicine buffer as before. All enzymes were expressed as recombinant proteins with N-terminal histidine tags using plasmid pET28a (Novagen). *Rhodospirillum rubrum* and *R. eutropha* RubisCOs were purified as described previously [66]. Following a similar procedure, PRK and PRI were purified by Ni–NTA affinity chromatography. The PRK gene was obtained from *Synechococcus* sp. PCC 7942 [67]. The genes encoding RubisCO and PRI enzymes were amplified from *R. eutropha* using appropriate primers from strain H16 (ATCC 17699).

### Transmission electron microscopy (TEM) imaging

Images were obtained with a Technai G2 Spirit instrument operating at 80 kV. Samples were freshly diluted in Bicine buffer. Drops (10 μL) of the sample solution in Bicine buffer were applied to carbon coated copper grids (Ted Pella, Inc.) for 2 min before removing the excess solution with filter paper. Images of Ni–NTA Nanogold^®^ particles (Nanoprobes, Inc.) bound to RubisCO or PRK, which were subsequently associated with the nanostructures, were obtained without the need for prior staining. Nanogold particles were pre-bound to histidine-tagged proteins by following the procedures described elsewhere (Nanoprobes, Inc.). Ni–NTA Nanogold^®^ particles mixed with just the nanostructures in the absence of histidine-tagged proteins provided evidence for the absence of non-specific association. For samples prepared without nanogold particles, uranyl acetate was used to stain the negatively-charged groups. To accomplish this, the grid containing the sample was floated on 10 μL drops of a 2% (w/v) uranyl acetate solution for 1 min before being imaged by TEM. Several replicates were imaged from at least 2 independent preparations to ensure that the imaging pattern was consistent for each sample.

### Enzyme assays and proteolysis

All enzyme activities were determined using end-point assays involving radioisotopes as described previously [64]. RubisCO activity measurements were performed via incorporation of NaH^14^CO_3_ into acid-stable [^14^C] 3-PGA, with a 1:1 stoichiometry of substrate to product. The kinetic constants *k*_cat_, *K*_c_, and *K*_o_ were determined from simultaneous assays performed in vials flushed with either 100% N_2_ or 100% O_2_, as per the procedure described elsewhere [68] with some modifications. Each reaction received an appropriate amount of enzyme (unbound or in nanostructure complexes) that would fix 2.5–5 nmol of CO_2_ per min, based on specific activity calculations. Unbound or nanostructure-bound RubisCO enzymes were pre-activated in identical volumes of Bicine buffer with 10 mM NaHCO_3_ and 1 mM dithiothreitol (DTT). Each 250-µL reaction mixture comprised of 50 mM Bicine-NaOH, pH 8.0, 10 mM MgCl_2_, 0.4 mM RuBP, and 8 levels of NaH^14^CO_3_, with corresponding CO_2_ concentrations ranging from ~10 to 120 µM for form I RubisCO samples and ~25–450 µM for form II RubisCO samples. Reactions were initiated with the addition of activated enzyme and terminated after 1 min with the addition of 200 µL of 3 M formic acid in methanol. The samples were dried in an oven at 80 °C overnight, products re-dissolved in 250 µL 0.25 M HCl, and counted with 5 mL of EcoScint H scintillation cocktail (National Diagnostics). Results were plotted using Sigma Plot 12.0. The *k*_cat_ and *K*_c_ values were derived from Michaelis–Menton plots of data from assays performed anaerobically, i.e., under 100% N_2_. The *K*_o_ [or *K*_i_ (O_2_)] values were obtained with data from parallel assays performed in the presence (1.23 mM) and absence (0 mM) of O_2_. The *k*_cat_ values for RubisCOs were calculated by noting the amount of free enzyme added to the nanostructures for forming the complexes because it was not possible to accurately determine the protein concentration within these complexes. The CO_2_/O_2_ specificity factor measurements involved the incorporation of [^3^H]-radioactivity from [1-^3^H]-RuBP into either a molecule of 3-PGA (carboxylation reaction) or a molecule of 2-phosphoglycolate (2-PG) (oxygenation reaction). These assays were performed with unbound or nanostructure-bound RubisCO (~30–50 µg) under saturating O_2_ concentrations (1.23 mM) with 5 mM NaHCO_3_ in Bicine buffer. The 400-µL reactions were initiated with the addition of [1-^3^H] RuBP (0.65 mM), and incubated at 23 °C for 1 h. The reactions were terminated by injecting 20 µL of 40 mM sodium borohydride. After 15 min, the excess sodium borohydride in the reaction was consumed with the addition of 20 µL of 160 mM glucose. After an additional 15 min, the reaction mixture was diluted with the addition of 1 mL of distilled water. The samples were then de-proteinated using Amicon Ultra Centrifugal filters (50,000 MWCO, Millipore). Reaction products present in 1-mL reaction mixtures were separated using an HPLC system (Shimadzu) on a 1-mL MonoQ column (GE Healthcare), and detected with an in-line scintillation counter (IN/US β-Ram). The ratio of areas in the 3-PGA and 2-PG peaks (corresponding to the *V*_c_/*V*_o_ ratio), was used to calculate the specificity values in each case. [1-^3^H] RuBP was synthesized and purified as described elsewhere [69].

For measuring the flux through the pathways, the respective enzymatic activities were coupled to the RubisCO reaction (i.e., by following the incorporation of ^14^C-label from NaH^14^CO_3_ into stable 3-PGA) with the addition of ATP and ribose-5-phosphate (R-5-P) (pathway 1), or with the addition of ATP, NADH, and glucose (pathway 2). Pathway 1 reactions comprised of 3.2 mM R-5-P, 6 mM ATP, 20 mM NaH^14^CO_3_, and approximately 3–40 µg of each pathway enzyme (unbound or in nanostructure complexes) in Bicine buffer. Pathway 2 reactions comprised of 100 mM glucose, 2 mM ATP, 2 mM NADH, 20 mM NaH^14^CO_3_, 2 mM DTT, and approximately 3–50 µg of each pathway enzyme (unbound or in complex with nanostructures).

For the determination of proteolytic susceptibilities, the nanostructure-RubisCO complexes were incubated with fixed amounts of subtilisin (molar ratios of 1 subtilisin per 2000 RubisCO molecules or 1 per 667 PRK molecules) for various times, followed by the addition of PMSF to arrest proteolysis [70]. The samples were immediately placed on ice and residual RubisCO or PRK activity was measured. PRK activity was measured by following the incorporation of NaH^14^CO_3_ into acid-stable [^14^C] 3-PGA, in coupled reactions with an excess of RubisCO. For each enzyme, the recovered activity percentages were plotted as a function of incubation time with subtilisin.

## Additional file

**Additional file 1: Figure S1.** Inhibition of RubisCO activity with monomeric compounds **A**, **B**, and **C**. **Figure S2.** TEM images of nanogold-labeled histidine-tagged RubisCO and PRK enzymes associated with nanostructures. **Figure S3.** HPLC fractionation profiles of 3H-labeled carboxylation-specific 3-PGA and oxygenation-specific 2-PG measured from samples with unbound and nanostructure-form I RubisCO complex. **Figure S4.** Schematic showing steps involved in the synthesis of compound **C**. **Figure S5.** Purity of compound **C**. **Table S1.** Effect of washing nanostructure-RubisCO complexes with buffer in the presence or absence of salt. **Table S2.** RubisCO activity recoveries obtained with varying concentrations of nanostructures. **Table S3.** Activities of individual enzymes in three-enzyme nanostructure complexes. **Table S4.** Combined activity of PRK and RubisCO enzymes present in the same nanostructure complex, or in mixtures with separate single-enzyme nanostructures.

## Abbreviations

RubisCO: ribulose 1,5-bisphosphate carboxylase/oxygenase
RuBP: ribulose 1,5-bisphosphate
R-5-P: ribose-5-phosphate
Ru-5-P: ribulose-5-phosphate
CBB cycle: Calvin–Benson–Bassham cycle
CA: carbonic anhydrase
BSA: bovine serum albumin
TEM: transmission electron microscopy
PRI: phosphoriboseisomerase
PRK: phosphoribulokinase
3-PGA: 3-phosphoglyceric acid
2-PG: 2-phosphoglycolate
NADH: reduced nicotinamide adenine dinucleotide
ATP: adenosine triphosphate
*K*_c_: Michaelis constant (*K*_m_) for CO_2_
*K*_o_: Michaelis constant for oxygen
*K*_RuBP_: Michaelis constant for RuBP
*V*_c_: *V*_max_ value for carboxylation
*V*_o_: *V*_max_ value for oxygenation
*Ω*: RubisCO’s CO_2_/O_2_ specificity factor (*V*_c_ *K*_o_/*V*_o_ *K*_c_)
Fmoc: fluorenylmethyloxycarbonyl
DMF: dimethyl formamide
DCM: dichloromethane
TFA: trifluoroacetic acid
HPLC: high-performance liquid chromatography
ESI–TOF: electrospray ionization time-of-flight
DIPEA: N,N-diisopropylethylamine
Ni–NTA: nickel-nitrilotriacetic acid
PMSF: phenylmethane sulfonyl fluoride
