# Supplementary material for: Synthetic CO2-fixation enzyme cascades immobilized on self-assembled nanostructures that enhance CO2/O2 selectivity of RubisCO
Source: Biotechnol Biofuels. 2017 Jul 6;10:175. doi: 10.1186/s13068-017-0861-6 (PMC5501267; doi:10.1186/s13068-017-0861-6)
Supplement: Supplementary file 1 — Additional file 1: Figure S1. Inhibition of RubisCO activity with monomeric compounds A, B, and C. Figure S2. TEM images of nanogold-labeled histidine-tagged RubisCO and PRK enzymes associated with nanostructures. Figure S3. HPLC fractionation profiles of 3H-labeled carboxylation-specific 3-PGA and oxygenation-specific 2-PG measured from samples with unbound and nanostructure-form I RubisCO complex. Figure S4. Schematic showing steps involved in the synthesis of compound C. Figure S5. Purity of compound C. Table S1. Effect of washing nanostructure-RubisCO complexes with buffer in the presence or absence of salt. Table S2. RubisCO activity recoveries obtained with varying concentrations of nanostructures. Table S3. Activities of individual enzymes in three-enzyme nanostructure complexes. Table S4. Combined activity of PRK and RubisCO enzymes present in the same nanostructure complex, or in mixtures with separate single-enzyme nanostructures. [file 13068_2017_861_MOESM1_ESM.docx]

Additional file 1

**Synthetic CO_2_-fixation enzyme cascades immobilized on self-assembled nanostructures that enhance CO_2_/O_2_ selectivity of RubisCO**

Sriram Satagopan^1†^, Yuan Sun^2†^, Jon R. Parquette^2*^, and F. Robert Tabita^1*^

^1^Department of Microbiology, The Ohio State University, Columbus, Ohio 43210

^2^Department of Chemistry and Biochemistry, The Ohio State University, Columbus, Ohio 43210

† - These authors contributed equally to the study

*To whom correspondence should be addressed: F. Robert Tabita, Department of Microbiology, The Ohio State University, 484 West 12^th^ Avenue, Columbus, OH 43210-1292; Phone: +1-614-292-4297; Fax: +1-614-292-6337; E-mail: tabita.1@osu.edu

*To whom correspondence should be addressed: Jon R. Parquette, Department of Chemistry and Biochemistry, The Ohio State University, 100 West 18^th^ Avenue, Columbus, OH 43210-1185; Phone: +1-614-292-5886; E-mail: parquette.1@osu.edu

**
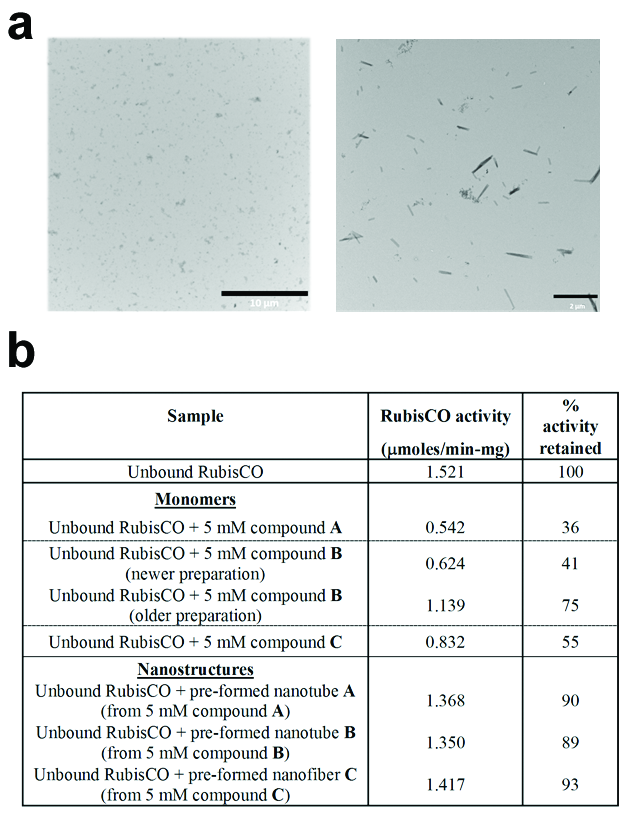
**

**Fig. S1** Inhibition of RubisCO activity with monomeric compounds **A**, **B** and **C**. **a** TEM images indicated that the older preparation of compound **B** had a significant proportion of pre-formed nanotubes (right) relative to a newer preparation (left). **b** Form II RubisCO activity measurements indicated that the older preparation of monomer compound **B** was less inhibitory relative to a newer preparation. Activity recovery calculations presented here are relative to the free unbound RubisCO.

**
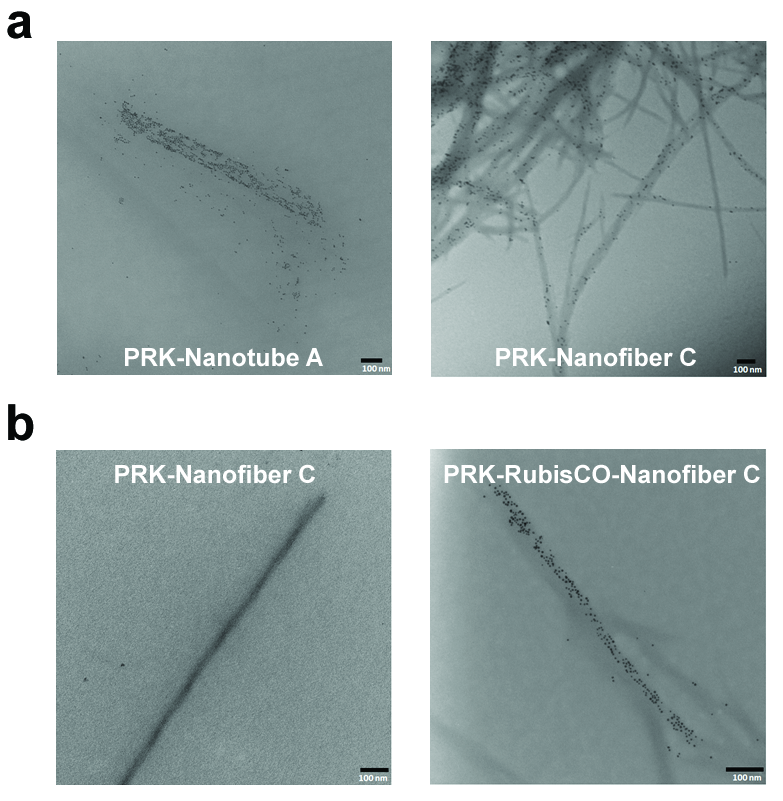
**

**Fig. S2** TEM images of Nanogold-labeled histidine-tagged proteins associated with nanostructures. **a** Images of nanogold-labeled PRK assembled into either a nanotube (left) or a nanofiber (right). **b** Images of a 1.8-nm nanogold-labeled PRK in nanofiber **C** (left) and a composite image of nanofiber **C** with 1.8-nm nanogold-labeled PRK and 5-nm nanogold-labeled RubisCO (form I) (right).


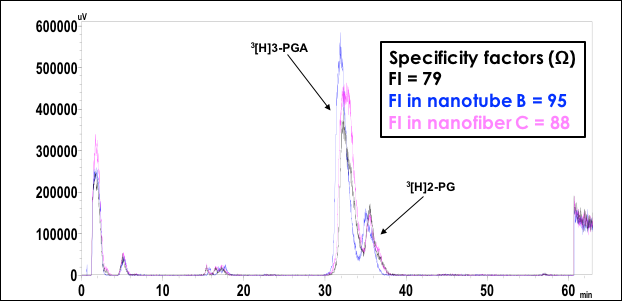


**Fig. S3** The CO_2_/O_2_ specificity factor (Ω) of the nanostructure-bound form I *R. eutropha* RubisCO is elevated. HPLC fractionation of ^3^H-labeled carboxylation-specific 3-PGA and oxygenation-specific 2-PG on an ion-exchange column for *R. eutropha* form I RubisCO that was free (*black*) or associated with either nanotube **B** (*blue*) or nanofiber **C** (*magenta*). The y axis indicates the signal (in μvolts) detected by an in-line radioactivity detector (LabLogic Systems, Inc.), which is proportional to the amount of [^3^H]-radioactivity present in the sample. Data is representative of profiles obtained from three independent experiments with separate nanostructure-enzyme preparations.

**
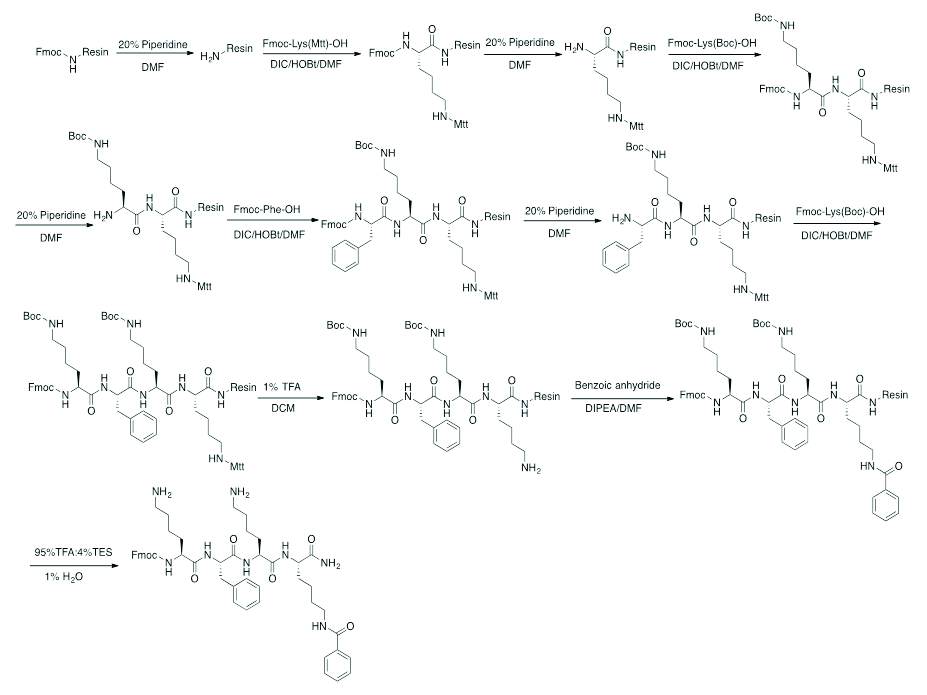
**

Fig. S4 Schematic showing the steps involved in the synthesis of Fmoc-KFKK(Bz) (compound C).

***
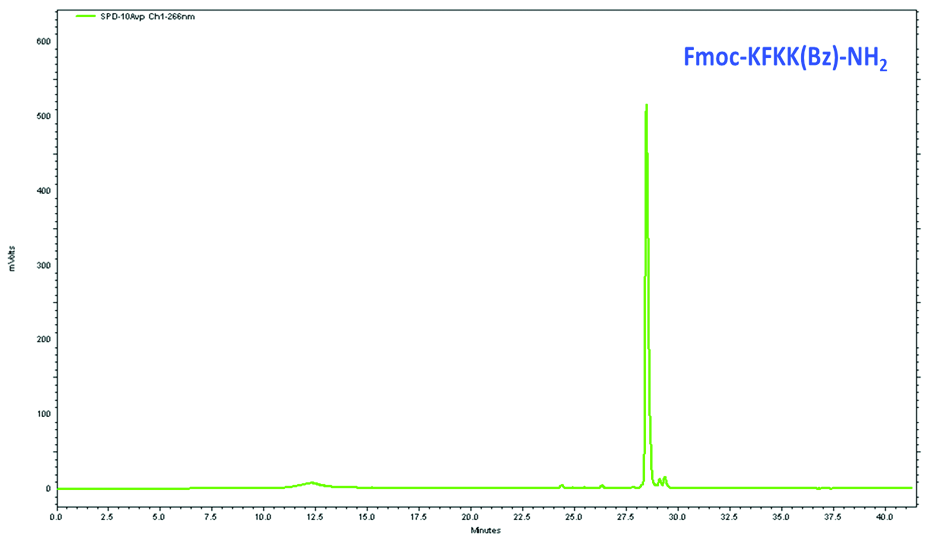
***

**Fig. S5** Purity of Fmoc-KFKK(Bz) (compound **C**). HPLC trace of compound **C**, eluted with Acetonitrile/Water 0.1% TFA: 10/90 to 100/0 over 45 min). The units for the y-axis (millivolts) indicates the signal detected by the machine, which is proportional to the absorbance at 266 nm.

**Table S1** Effect of washing nanostructure-RubisCO complexes with buffer in the presence or absence of salt. *R. eutropha* form I (FI) or *R. rubrum form* II (FII) RubisCOs were each assembled with nanotube A or nanofiber C. After isolating the complexes using ultracentrifugation, aliquots of the nanostructure-RubisCO complexes were re-suspended into Bicine buffer in the presence or absence of 300 mM NaCl. Differentially-treated complexes were re-isolated using ultracentrifugation, re-suspended in Bicine buffer (with no salt) and RubisCO activities were measured. Percentage activities were calculated relative to the activities of the respective unbound RubisCOs.

| **Sample** | **Wash** | **Specific activity** | **% activity** |
| --- | --- | --- | --- |
|  |  | **(μmoles/min-mg RubisCO)** | **(relative)** |
| Unbound FI | None | 1.393 | 100 |
| FI in nanotube A | None | 0.291 | 21 |
|  | Buffer | 0.360 | 26 |
|  | Buffer/NaCl | 0.392 | 28 |
| FI in nanofiber C | None | 0.993 | 71 |
|  | Buffer | 0.922 | 66 |
|  | Buffer/NaCl | 0.901 | 65 |
| Unbound FII | None | 1.375 | 100 |
| FII in nanotube A | None | 0.401 | 29 |
|  | Buffer | 0.427 | 31 |
|  | Buffer/NaCl | 0.387 | 28 |
| FII in nanofiber C | None | 0.545 | 40 |
|  | Buffer | 0.488 | 35 |
|  | Buffer/NaCl | 0.486 | 35 |

**Table S2** RubisCO activity recoveries obtained with varying concentrations of nanostructures. Nanostructure-RubisCO complexes were assembled by adding *R. eutropha* form I (FI) or *R. rubrum* form II (FII) RubisCOs (at 1 mg/ml final concentration) to varying amounts of nanotube A or nanofiber C preparations. After isolating the complexes using ultracentrifugation, they were re-suspended into Bicine buffer and RubisCO activities were measured. Percentage activities were calculated relative to the activities of the unbound form I or form II RubisCOs. Data is representative of dosage experiments that employed varying sets of nanostructure concentrations.

| **Sample** | **Starting monomer concentrations** | **Monomer equivalents in final sample** | **Specific activity** | **% activity** |
| --- | --- | --- | --- | --- |
|  | **(mM)** | **(mM)** | **(μmoles/min-mg RubisCO)** | **(relative)** |
| Unbound FI |  |  | 1.597 | 100 |
| FI in nanotube A | 20 | 4 | 0.411 | 26 |
|  | 10 | 2 | 0.616 | 39 |
|  | 1 | 0.2 | 0.519 | 32 |
| FI in nanofiber C | 10 | 4 | 0.514 | 32 |
|  | 5 | 1 | 0.679 | 43 |
|  | 1 | 0.2 | 0.620 | 39 |
| Unbound FII |  |  | 1.189 | 100 |
| FII in nanotube A | 20 | 4 | 0.566 | 48 |
|  | 10 | 2 | 0.276 | 23 |
|  | 1 | 0.2 | 0 | 0 |
| FII in nanofiber C | 10 | 4 | 0.188 | 16 |
|  | 5 | 1 | 0.573 | 48 |
|  | 1 | 0.2 | 0.143 | 12 |

**Table S3** Activities of individual enzymes in three-enzyme nanostructure complexes. **a** PRI activities measured using a coupled assay supplemented with excess PRK and RubisCO (form I, FI, and form II, FII). **b** PRK activities measured using a coupled assay supplemented with excess PRI and RubisCO. **c** RubisCO activities measured using RuBP as the starting substrate. Data is representative of results from two independent nanostructure-enzyme complex preparations. Activities retained were calculated relative to the activity of unbound enzyme sample.

**
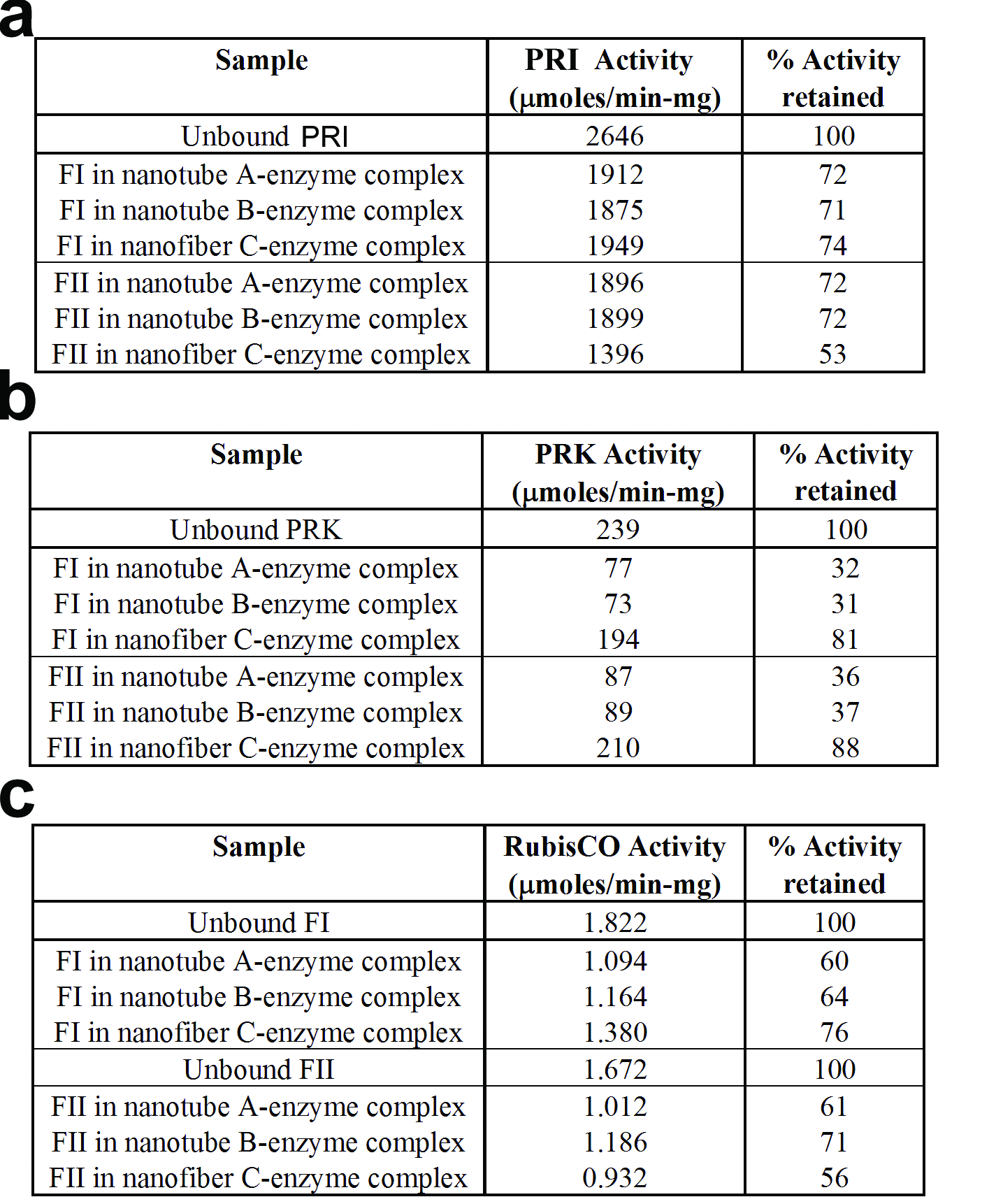
**

**Table S4** Combined activity of PRK and RubisCO enzymes present in the same nanostructure complex, or in mixtures with separate single-enzyme nanostructures. *R. eutropha* form I (FI) RubisCO and PRK enzymes were assembled into either separate single-enzyme nanostructures or into the same nanostructures (control). Combined activities were measured using a coupled assay by mixing nanostructure-RubisCO and nanostructure-PRK complexes in the ratio 20:5. A similar ratio of enzymes was used in the assembly of a single nanostructure with both enzymes for each control. Percentage recoveries are indicated relative to the respective control samples. Data is representative of two independent experiments that gave similar results.

| **Sample** | **Specific activity** | **% activity** |
| --- | --- | --- |
|  | **(μmoles/min-mg RubisCO)** | **(relative)** |
| FI & PRK in nanotube A (control) | 1.46 | 100 |
| FI in nanotube A + PRK in nanotube A | 0.40 | 27 |
| FI in nanotube A + PRK in nanofiber C | 0.41 | 28 |
| FI & PRK in nanofiber C (control) | 2.46 | 100 |
| FI in nanofiber C + PRK in nanotube A | 1.84 | 75 |
| FI in nanofiber C + PRK in nanofiber C | 1.94 | 79 |
